# Current Hospital Topics

**Published:** 1906-11-24

**Authors:** 


### HOSPITAL ADMINISTRATION.

### CONSTRUCTION AND ECONOMICS.

### CURRENT HOSPITAL TOPICS.

#### Shakespeare in the Hospitals.

Sir Riley Lord has started a new method of raising funds for the Royal Victoria Infirmary in Newcastle. He invited Mr. W. R. Yardley to deliver a lecture on "Shakespeare, the greatest Englishman," profusely illustrated with limelight views, in the out-patients' hall of the Royal Victoria Infirmary. The idea caught the public fancy, and there was a large and enthusiastic audience. The Newcastle Infirmary has benefited directly from the money thus raised, and indirectly by extending the interest in the work of this hospital to many people who otherwise might never have taken any part in the business. Why should not every out-patient hall be similarly utilised, during the winter months, to the no small advantage of the hospitals and the citizens alike?

#### Royal South Hants Hospital.

Colonel Willan, J.P., has resigned the chairmanship of this hospital which he has held for seven years. During these years this hospital has greatly advanced in reputation and efficiency. It possesses at the present time some of the most attractive and well designed wards to be met with in the provinces. We understand that Colonel Willan has resigned in consequence of the desirability of his wintering abroad. We can assure him that the work which has been done at Southampton during his chairmanship, with the co-operation of Miss Mollett, the very able matron, has so impressed us, that we have never failed to recommend visitors to this country to go to Southampton, in order to see this hospital as a good example of what may be accomplished in the way of modernising an old hospital, where ability and brains have combined to secure a wise expenditure of the funds in hand.

#### From House to House.

For a lengthened period it has been the practice in the city of Edinburgh for a systematic canvass from house to house to be made each year on behalf of the Royal Infirmary. The plan is for the hospital's representative to call with a book, in which each giver is asked to sign his name with the amount given, and these books are produced to each householder from year to year. When the old signature is seen the Scotsman pays promptly and pleasurably too. Why cannot this system be carried out in other cities or portions of them? At Norwich a house-to-house collection has been made under the direct supervision of the Mayoress, Mrs. Edward T. Bordman, by 373 ladies and 11 gentlemen. The city was divided into 34 districts, and £415 was obtained from house-to-house collections. We congratulate the Mayoress and the citizens of Norwich upon a new departure which may in a few years prove the most fruitful source of revenue possessed by the Norfolk and Norwich Hospital.

#### City and County Hospital Conferences.

In the West Riding efforts are being made to bring the boards of management of various hospitals into closer relationship with each other. It is felt that periodical conferences of the representatives of such boards would elicit information which must prove helpful to all. In the West Riding there are more than eighty public hospitals for the reception of infectious diseases including smallpox. At the first conference held on the 13th instant at Leeds it was determined to admit representatives of the county borough hospitals and to invite the county boroughs to send representatives to the conference in January, 1907. The proceedings on the 13th instant were quite informal and preliminary. If these conferences are to succeed the secretary, Mr. J. C. Jackson, chartered accountant of Leeds, will have to obtain the assistance of a small committee of medical officers and chairmen, who must formulate the subjects to be dealt with at the next conference. The most practical way to secure the results aimed at would seem to be to adopt the question-box method, which makes similar gatherings so interesting at times in the United States. Those interested are invited to send in to the secretary questions on practical points to which they desire to obtain an authoritative answer. The chairman is selected for his general knowledge of the subjects likely to arise, and he arranges the order in which the questions are read out and debated. In this way many practical reforms have been secured, whilst the interest of meetings never flags.

#### The Association of Hospital Superintendents.

This association which held its annual meeting this year at Buffalo is to be re-organised with a view to increasing its effectiveness. In future there are to be permanent standing reportorial committees, which will systematically review the various branches of hospital work in the United States. The work of these committees will enable a comprehensive statement of the progress made each year to be regularly forthcoming at the annual meetings. A further movement is progressing in the United States which it is interesting to note as an example which may be followed with possible advantage in this country. We refer to the organisation of local hospital conferences in the greater cities of America. In New York, after two years' steady work a committee has succeeded in welding together an organisation representing nearly forty voluntary hospitals, and a scheme has just been adopted for carrying on the work. It will be interesting to note how far this laudable effort proves successful in practice. It must succeed if sufficient interest, zeal and literary and critical ability can be developed among the members of the organisation. Hospitals nowadays form so important a portion of the economy of great cities, that the advantages of a system of co-ordination between all the voluntary and public hospitals, with a view to securing an administration which will best promote the interests of all concerned, cannot be doubted by any intelligent and wise administrator. Of course this proposal represents a great step in advance, but we hope to see it successfully taken within the next few years.

